# The ecological importance of habitat complexity to the Caribbean coral reef herbivore *Diadema antillarum*: three lines of evidence

**DOI:** 10.1038/s41598-021-87232-9

**Published:** 2021-04-30

**Authors:** M. D. V. Bodmer, P. M. Wheeler, P. Anand, S. E. Cameron, Sanni Hintikka, W. Cai, A. O. Borcsok, D. A. Exton

**Affiliations:** 1 Operation Wallacea, Wallace House, Old Bolingbroke, Spilsby, Lincolnshire, PE23 4EX UK; 2 School of Environment, Earth and Ecosystem Sciences, The Open University, Walton Hall, Milton Keynes, MK7 6AA UK; 3 School of Life Sciences, College of Science, University of Lincoln, Brayford Way, Lincoln, LN6 7TS UK; 4 University College Dublin, Belfield, Brayford Way, Dublin, 4 Ireland; 5 Department of Life Sciences, Imperial College London, London, SW7 2BX UK; 6 Tela Marine Research Centre, Honduras Shores Plantation, Tela, Atlantida Honduras

**Keywords:** Restoration ecology, Conservation biology, Ecosystem ecology, Restoration ecology, Tropical ecology

## Abstract

When Caribbean long-spined sea urchins, *Diadema antillarum*, are stable at high population densities, their grazing facilitates scleractinian coral dominance. Today, populations remain suppressed after a mass mortality in 1983–1984 caused a loss of their ecosystem functions, and led to widespread declines in ecosystem health. This study provides three lines of evidence to support the assertion that a lack of habitat complexity on Caribbean coral reefs contributes to their recovery failure. Firstly, we extracted fractal dimension (D) measurements, used as a proxy for habitat complexity, from 3D models to demonstrate that urchins preferentially inhabit areas of above average complexity at ecologically relevant spatial scales. Secondly, controlled behaviour experiments showed that an energetically expensive predator avoidance behaviour is reduced by 52% in complex habitats, potentially enabling increased resource allocation to reproduction. Thirdly, we deployed a network of simple and cost-effective artificial structures on a heavily degraded reef system in Honduras. Over a 24-month period the adult *D. antillarum* population around the artificial reefs increased by 320% from 0.05 ± 0.01 to 0.21 ± 0.04 m^−2^ and the juvenile *D. antillarum* population increased by 750% from 0.08 ± 0.02 to 0.68 ± 0.07 m^−2^. This study emphasises the important role of habitat structure in the ecology of *D. antillarum* and as a barrier to its widespread recovery.

## Introduction

Coral reefs are among the most valuable and vulnerable ecosystems on the planet^1,2^. Loss of herbivory, largely driven by disease and the widespread unsustainable harvesting of fish and echinoids in small-scale fisheries^3,4^, has many negative impacts on coral reefs, but it is the associated macroalgae overgrowth that poses the greatest threat^5^. In the absence of herbivores, structurally complex, three-dimensional hard corals are replaced by structurally simple two-dimensional macroalgae^6,7^, which reduces habitat availability and drives decreases in species richness and abundance^8^. Species richness begets functional redundancy and response diversity, which confers resilience^9,10^ and has a stabilising effect on ecosystem function^11^.

In comparison to the Indo-Pacific, the Caribbean has just 28% and 14% of the diversity of fishes and corals respectively^12^, and its reefs are consequently less resilient than other global hotspots^13,14^. This relative lack of diversity means that the echinoid herbivore, *Diadema antillarum*, is historically considered among the most important macroalgae grazers in the Caribbean^15–17^, and its relative importance likely increased since mass overfishing reduced abundances of key herbivorous fish^18^. Numerous modelling and experimental studies indicate that *D. antillarum* ecosystem functions are activated at densities of 0.6–1.0 individuals m^−2^ ^19–21^, and high-density populations can consume the entire daily growth of macroalgae^22^. The negative correlation between *D. antillarum* population density and macroalgae cover is well documented^23^ and is closely associated with a positive correlation between echinoid density and hard coral cover^15,24–28^.

In the early 1980s, an unknown water-borne pathogen reduced *D. antillarum* populations by 95–100% as it spread throughout the Caribbean^25,29–31^. Historical records of *D. antillarum* population densities are inconsistent, but the impacts of, and subsequent recovery from, the disease have been well documented in Panama. Here, densities were reduced from an average of 3.5 m^−2^ ^32^, to 0.0001 m^−2^ ^29^, and two decades later populations had recovered to just 6.5% of their pre-mortality levels^33^. This pattern of population destruction followed by a lack of recovery has been observed across the entire extent of the region from Barbados^34^, Venezuela^35^, and Curacao^36,37^ in the south, to the Florida Keys^16^ in the north. The functional extinction of *D. antillarum* led to Caribbean-wide macroalgae increases of up to 50%^38^ and is undoubtedly a contributing factor to the 80% coral loss observed in the Caribbean from 1970 to 2000^39^, and the continuing decline to an average cover of just 16.3% in 2012^7^.

*D. antillarum* restoration is a priority, but first the mechanisms by which populations are being supressed must be identified and removed^19^. Echinoids are density-dependent external fertilisers and reproductive success correlates with adult population density^40,41^. At low density, large nearest-neighbour distances reduce the probability of fertilisation success, therefore reductions in population density associated with the mass-mortality likely created an Allee effect that has prevented recovery^42^. This effect may be continually reinforced by a positive feedback loop that was established after the die-off; loss of *D. antillarum* ecosystem functions caused declines in structurally complex hard coral cover, which reduced the availability of their own habitat^28^. In turn, this increased the average nearest-neighbour distance between individuals^40^ and exacerbated the Allee effect ultimately driving further declines in adult population density. The negative impact of this Allee effect is likely compounded by the poor success of juvenile recruits who are unable to seek shelter in the spiny canopy of densely aggregated adults, or within the complex framework of the reef which has flattened considerably since the 1980s^8,43^.

Numerous correlative studies have shown a positive relationship between *D. antillarum* population density and habitat structure. These studies have elucidated an important ecological driver of *D. antillarum* dynamics, and hypothesise there are two mechanisms by which complex habitats likely facilitate survival of larger populations; by increasing recruitment, and providing predation refugia that facilitate juvenile survival into adulthood^28,44–46^. *D. antillarum* reintroduction may therefore be successful, if coupled with the artificial augmentation of 3D habitat complexity, although our current understanding is based largely on correlative analyses and urgently needs to be supported by experimental evidence.

Here we extend our understanding of the importance of habitat complexity by exploring its relationship with *D. antillarum* population structure and behaviour using a more experimental approach. We present three independent lines of evidence highlighting the ecological importance of habitat complexity to the survival, distribution and recovery of *D. antillarum* on Caribbean coral reefs. First, we use structural complexity (fractal dimension) data extracted from 3D models of the underlying reef architecture to show that individuals from a low-density population are found in areas of reef with disproportionately higher habitat complexity than the background average at an ecologically relevant spatial scale. We hypothesise that at the 5–15 cm spatial scale, *D. antillarum* exhibit a preference for higher complexity habitat because their test diameter can be up to 10 cm^47^, and they must also have room for their articulated spines. We then use an ex situ lab-based experiment to address the hypothesis that *D. antillarum* demonstrate behavioural changes in the presence of enhanced habitat complexity that reduce energetic requirements associated with predator avoidance and thus improve survival potential. Finally, we use our findings to justify the deployment of a network of simple and cost-effective artificial reefs, designed to provide optimal habitat complexity to both juvenile and adult *D. antillarum*, on a highly degraded reef system. Our results support the hypothesis that enhancement of reef structure can augment *D. antillarum* populations as we report significant increases in densities over a 24-month period relative to nearby control reefs.

## Methods

### Study sites

We investigated the importance of habitat structure to *D. antillarum* population dynamics using two in situ field studies and an ex -situ lab-based experiment. The in situ studies allowed us to explore the impacts of habitat structure on *D. antillarum* population dynamics, whilst the ex-situ study enabled elucidation of the mechanisms that may drive the relationships observed in the field.

These studies took place on the reefs of Utila and Tela Bay, at the southern end of the Mesoamerican Barrier Reef System (MBRS) in Honduras. Utila is one of the Bay Islands of Honduras located approximately 40 km from the mainland and it is a popular tourist destination. Tela Bay is home to a diverse range of marine ecosystems, including Banco Capiro, which has a hard coral and macroalgae cover of 62% and 7% respectively^48^, making it one of the healthiest reef systems in the Caribbean. In 2018, an 822.6 km^2^ area of the bay was afforded protection by the Honduran government as part of the El Refugio de Vida Silvestre Marino de Tela.

### In situ habitat preferences of *D. antillarum*

We studied *D. antillarum* habitat complexity preferences on reefs around the island of Utila, Honduras, which is home to an extensive fringing reef system^49^. With mean scleractinian coral cover of 12–22%, and *D. antillarum* populations varying from 0.17 m^−2^ at 2 m depth to just 0.01 m^−2^ at 10 m depth^28^, it represents a ‘typical’ contemporary Caribbean reef with little to no post-mortality recovery of *D. antillarum*^50^.

Habitat complexity was measured within 2 × 2 m quadrats placed randomly on six reef sites along the south shore of the island at depths of 8–10 m. The presence/absence of *D. antillarum* was recorded in each quadrat; 35 quadrats without urchins were assessed and a further five urchin-containing-quadrats were sampled to allow complexity differences to be elucidated. The uneven sample sizes, a result of the dearth of *D. antillarum* on study reefs, were accounted for in the statistical analyses.

#### Collection and production of 3D models

Advancements in 3D modelling technologies have enabled researchers to move away from outdated and inaccurate methodologies used for assessing habitat complexity, e.g. Risk’s chain method for quantifying rugosity^51^. Habitat complexity measurements, in the form of fractal dimension (D), can be extracted from 3D models at different spatial scales, allowing researchers to gain a better understanding of the importance of 3D architecture to their study organism.

Underwater photogrammetry using structure-from-motion is now a well-established approach used to quantify complexity on coral reefs^52–55^. Here, we followed an existing method for footage collection, model construction and analysis^55^. In summary, 3D models were built based on video scans of the quadrats, filmed from a birds-eye perspective with a single camera (GoPro Hero 4 Black) held approximately 50 cm above the substrate. The quadrat was swept in a lawnmower pattern, ensuring a minimum overlap of 25 cm between passes; essential for successful image alignment and model construction. Each video was approximately 3 min long.

Videos were converted to still images using QuickTime Player v.7.6.6 (QuickTime 1989–2010) at an extraction rate of 3 frames per second which generated 450 to 540 still images per quadrat. These images were uploaded to PhotoScan Pro v.1.3.2 (Agisoft PhotoScan Professional 2017) and 3D models constructed. Resultant PhotoScan files (.psx) were converted to object files (.obj) and imported into Rhinoceros 3D 5.3.2 (Rhinoceros 1993–2017) for analysis.

#### Quantifying habitat complexity using fractal dimension

We quantified habitat complexity using fractal dimension (D), which measures how complexity changes between two defined resolutions^56^, making it ideal when focusing on a single study organism as it allows complexity which is ecologically relevant to that species to be quantified in isolation. It returns a value between 2 and 3, with a higher number indicating greater complexity, and was calculated using a published Python script^55^.

*D. antillarum* have fully articulated spines^57^ therefore their ability to fit into a crevice is determined by their test diameter. Crevices in the reef provide echinoids with predation refugia^16^ but only if the gap between their test and sides of the crevice prevents predators from entering. To identify spatial scales that are ecologically relevant to local *D. antillarum* populations with regards to predator avoidance, we measured the test diameter of adult *D. antillarum* at our two study locations in Utila (*n* = 139) and Tela Bay (*n* = 100). Using SCUBA, we searched the reef for adult urchins and coerced them from their crevice using a 50 cm length of PVC pipe. The test diameter, excluding spines, of each individual was measured using a long-jaw calliper.

To assess variation in *D. antillarum* structural preferences at different spatial scales, we calculated D at the following resolutions: 1–5 cm, 5–15 cm, 30–60 cm and 60–120 cm. Test diameter measurements in Utila revealed that the 5–15 cm resolution was likely to be the most relevant to local *D. antillarum* because adult test diameters ranged from 21 to 90 mm. *D. antillarum* spines often measure > 30 cm but they are highly articulated, therefore an individual’s ability to occupy a crevice is determined by its width when the spines are folded over the test^57^; an individual with a test diameter of 21 mm will likely need a > 50 mm crevice and an individual with a test diameter of 90 mm will likely need a > 120 mm crevice. The five size categories were ultimately used to plot complexity signatures, providing a visual representation of how complexity changes across spatial scales between reef areas with and without urchins. This approach has recently been used successfully to demonstrate size-specific habitat associations of invasive Caribbean lionfish^58^.

### Ex situ impacts of structural complexity on *D. antillarum* predator avoidance behaviour (PAB)

The in situ habitat structure preference study elucidated that *D. antillarum* prefer complex habitats at the 5–15 cm spatial scale. We therefore conducted an ex situ lab-based study to try and identify the mechanisms that may drive this relationship.

*D. antillarum* predator avoidance behaviour (PAB) is defined as the number of long-defensive spines that move in response to a predatory threat, e.g. a shadow stimulus^48,59–62^. Decreased light intensity may signal the presence of a predator, and it causes the expansion of melanophores in the dermis, which induces a nervous signal that stimulates spine movement thus decreasing the efficacy of a predatory attack^63^. Bodmer et al.^48^ found that PAB of individuals collected from architecturally complex reefs was lower than those collected from structurally simple reefs. They suggested that complex habitats, by offering natural protection against predation, allow individuals to gain energetic benefits from reducing their reliance on PAB^59,60^. Here, we use the same approach as Bodmer et al.^48^ but go further by introducing varying levels of habitat structure into the experimental setup.

#### Setup of the trial tanks

**Figure 1:**
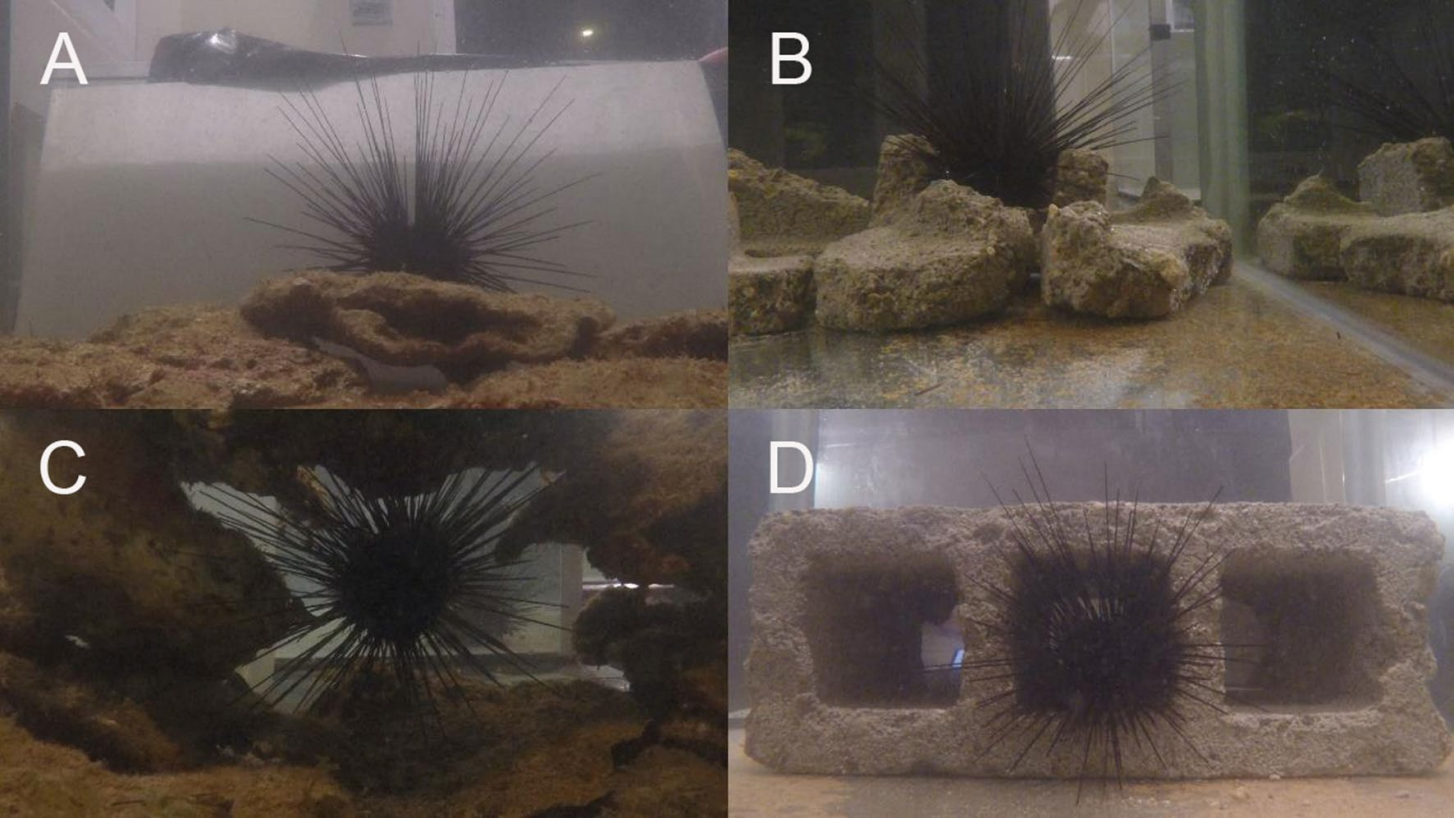
Panel photograph showing tank setup for different complexity treatments. Top left natural-material-low-complexity, top right = artificial-material-low-complexity, bottom left = natural-material-high-complexity, bottom right = artificial-material-high-complexity.

PAB was measured under three different complexity treatments by enriching trial tanks with either natural or artificial reef material. For ‘flat’ treatments no reef material was added to the tanks. For the ‘natural’ treatments, trial tanks were enriched with rubble collected from the reef; low-complexity-natural-material assays involved placing a single layer of reef rubble over the bottom of the tank, and high-complexity-natural-material assays were built off this low-complexity foundation, but were enhanced by the addition of ‘walls’ and a ‘roof’ in order to simulate a natural *D. antillarum* reef crevice (Fig. 1). During ‘artificial’ treatments, *D. antillarum* were provided with habitats made from concrete; low-complexity-artificial-material assays were created by breaking up a breezeblock and lining the bottom of the tank with the fragments, high-complexity-artificial assays involved placing a whole breezeblock in the tank (Fig. 1).

The location of the reef material in the tanks was assigned prior to each trial using a random number generator to control for the position effect. 50 black adult urchins were tested under 5 different experimental treatment combinations in a fully factorial design (n = 10 for each combination). Each urchin was used for a single trial only.

Each trial was carried out in one of three 300 L tanks containing fresh seawater which was replaced daily. Aquarium heaters (Aquael Easy Submersible Aquarium Heater 150w) were used to maintain trial tanks at 29.7 ± 0.3 °C. This control temperature was selected because throughout the trial period (May to August 2016) temperatures in the study area (longitude = 15.879404; latitude = − 87.515967) ranged from 28.58 to 31.68 °C and the mean SST was 29.87 °C (daily mean SD = ± 0.71 °C)^64^. Six trials were completed daily and the treatment combination in any given tank was randomised along with the treatment order to control for potentially confounding variables.

#### Collection of urchins

Urchins were collected from Banco Capiro, a large offshore reef system found ca. 8 km from the coast in Tela Bay, Honduras. With a mean hard coral cover of 62% at 10 m, and associated *D. antillarum* population densities of 2.6 m^−2^ ^28^, Banco Capiro may be one of the healthiest reefs found in the Caribbean today. Individuals were removed from the reef using a 50 cm length of PVC piping. Six specimens were collected each day, retained for a maximum of 24 h and returned to the site of their collection the following morning. Pseudoreplication was avoided by returning urchins to a different sub-site of the reef from where new specimens were collected. Prior to commencement of trials urchins were placed in 300 L glass holding tanks maintained at ambient SST in the laboratory for at least eight hours. Conservation of *D. antillarum* is a priority throughout the Caribbean, and so short acclimatisation periods were used to minimise the risk of mortality caused by experimentally induced stress.

#### Trial protocol and data generation

For each trial an individual *D. antillarum* was placed in the trial tank and allowed to settle for 30 min. Urchins naturally seek shelter, therefore, in treatments where it was available, they quickly moved towards the structure in the tank. After the settling period, a GoPro Hero 4 was set to record, placed in the tank and positioned to focus on the urchin.

Presence of a predator was simulated by creating a shadow over the tank^48^. Predator avoidance behaviour (PAB) was defined as the number of spines that move in response to the shadow stimulus as a percentage of the total number of long defensive spines visible in the video. This effectively standardises the measure between urchins and negates the problem that some long spines may be obscured by structures in the tank. Videos were analysed in a random order to avoid observer bias.

### Impacts of artificially augmenting habitat structure on *D. antillarum* population density

The results of the first two studies indicated that *D. antillarum* may prefer complex habitats because they provide predation refugia. We therefore deployed a network of artificial reefs (ARs) at La Ensenada, a shallow patch reef (3–10 m depth) located in Tela Bay, to see if augmentation of habitat structure could promote population recovery, and to assess the validity of ARs as a population restoration strategy. Initial diver observations indicated that La Ensenada is a degraded system with hard coral cover well below 10%, macroalgae cover up to 60%, and *D. antillarum* population densities significantly less than the 1 m^−2^ required for their ecosystem functions to be activated^20^.

#### Artificial reef design and deployment

In summer 2015, 30 experimental ARs were deployed, each constructed from 16 locally manufactured breezeblocks/cinder blocks (external dimensions: 41 × 20 × 20 cm) incorporating three holes of 10 × 10 cm diameter. Breezeblocks with these dimensions were chosen since (i) many AR initiatives fail because of high costs^65^, (ii) concrete has been identified as a suitable AR material^19,66^, (iii) they were readily available locally, (iv) our ex-situ PAB study demonstrated no effect of artificial (concrete) versus natural structure on urchin behaviour (see “Results” section) and (v) our survey of *D. antillarum* test diameters in Tela Bay suggested 10 × 10 cm diameter holes would provide suitable refugia for individuals at the lower end of the test size range (i.e. likely new recruits to our artificial reefs).

**Figure 2:**
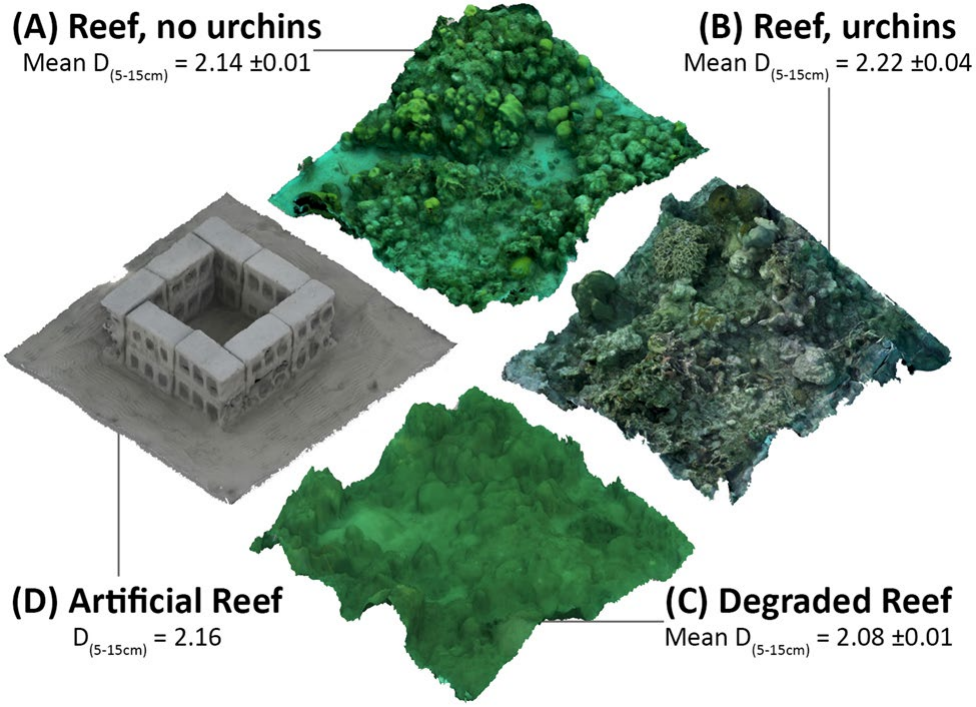
Three-dimensional images showing complexity differences between: (**A**) a 2 m × 2 m area of reef without urchins on Utila, (**B**) a 2 m × 2 m area of reef with urchins on Utila, (**C**) a 2 m × 2 m area of control reef in La Ensenada, (**D**) an artificial reef deployed at La Ensenada. D_(5–15 cm)_ = fractal dimension of the model at the 5–15 cm spatial resolution. Mean ± 1SE is reported for (**A**)–(**C**), but all artificial reefs are identical therefore variation in fractal dimension for (D) is not reported.

Ten ARs were constructed at each of three sub-sites; Palm View 1 (N 15.80337 W 087.43922), Palm View 2 (N 15.80336 W 8743955), and Becky’s Choice (N 15.80494 W 87.43955). ARs were separated by a minimum distance of 30 m, with lift bags used to safely lower materials from a support boat to the seafloor. Once all blocks had been lowered, the SCUBA team constructed the AR by creating a two-layered square structure on the benthos with holes facing outwards/inwards (Fig. 2).

#### Assessing population recovery

*D. antillarum* population assessments and benthic surveys were carried out around each AR immediately after deployment (summer 2015), after 12 months (summer 2016) and after 24 months (summer 2017). In each survey period, data were collected from ten 1 m^2^ quadrats that were randomly placed within a 5 m radius of the centre of the AR. In each quadrat, the abundance of *D. antillarum* was recorded along with the life-history stage of all individuals that were encountered; juveniles are identifiable by distinctive black and white striped spines^57^. Quadrats were also photographed and analysed for juvenile coral recruits, defined here as coral colonies < 40 mm in their longest dimension^23^, and for percentage cover of macroalgae using CoralPointCount (CPCe) software with 100 points randomly overlaid on each image. Percentage cover of mature scleractinian coral was not examined due to the relatively short (24 month) timeframe of this project meaning genuine changes were unlikely to be reliably detectable.

Control data were collected following Bodmer et al.^28^ to ensure that any observed inter-year differences were associated with AR deployment and not natural changes in the system. Randomly placed 50 × 2 m belt transects created a 100 m^2^ survey area in which *D. antillarum* and juvenile coral recruit abundances were recorded. Benthic percentage cover was calculated using point-intercept video transects analysed at 0.25 m intervals. Two transects were carried out on control reefs located at least 100 m away from any of the ARs at each sub-site (n = 6).

The methods used to assess the relative changes around the artificial and control reefs differed. Transects allowed us to survey a large area of the control reef to gain an accurate understanding of the background patterns of change, but they were not appropriate for assessing localised changes within 5 m of the AR structures. As a result, these data are not directly compared statistically.

### Statistical methods

All analyses were conducted using R v. 3.3.1^67^ and RStudio v0.99.903^68^.

#### Assessing in situ habitat preferences

The first part of this study used fractal dimension (D) as a proxy for structural complexity to explore the in-situ habitat preferences of *D. antillarum*. We assessed differences in D between quadrats inhabited by *D. antillarum* to those without, using Mann–Whitney U tests, which are robust to unbalanced survey designs. We conducted one test for each of the five spatial resolutions (1–5 cm, 5–15 cm, 30–60 cm and 60–120 cm), and adjusted the results for multiple comparisons using the Bonferroni method.

#### Assessing impacts of structure on *D. antillarum* predator avoidance behaviour (PAB)

Predator avoidance behaviour was evaluated as a function of reef complexity (flat, low, and high), and reef material (natural and artificial), and their interaction using a two-way analysis of covariance (ANCOVA). Post-hoc Tukey–Kramer analyses were used to assess pairwise differences in PAB among treatment combinations. Visual analysis of the residuals revealed that there were no influential outliers, the data were independent and normally distributed, and there was homogeneity in the variance, therefore parametric analyses were considered appropriate for these data.

#### Assessing the impacts of artificial reefs on *D. antillarum* population density and benthic composition

Changes in adult and juvenile *D. antillarum* population density within a 5 m radius of artificial reefs and on control reefs were assessed over a 3-year period (2015, 2016, 2017). Inter-year comparisons of juvenile coral recruit abundance, and macroalgae cover were also made. Separate analyses were conducted for each of these ecological variables, and analyses were also separated by reef type to account for differences in the way the data were collected on control reefs and around the artificial reefs. Shapiro–Wilk tests conducted on the residuals of each ecological variable by year revealed a non-normal distribution in most cases (Table 1), therefore we used non-parametric analyses. A series of ten Friedman tests, designed for use on datasets with one-way repeated measure designs, were used to assess changes in *D. antillarum* population density (adult, juvenile, and total), coral recruit abundance, and macroalgae cover between 2015, 2016 and 2017 (Table 2). Post-hoc Conover Iman tests^69^ were used to elucidate where observed differences were found; these were adjusted using the Bonferroni correction to account for the multiple pairwise comparisons.

**Table 1:** Data were separated by reef type (artificial or control) and analysis of the residuals of each ecological variable by year was conducted using a Shapiro–Wilk test for normality.

Reef type	Model	*W*	*p*
Artificial reef	Urchins_Adult ~ Year	0.71	< 0.05
Artificial reef	Urchins_Juvenile ~ Year	0.58	< 0.05
Artificial reef	Urchins_Total ~ Year	0.66	< 0.05
Artificial reef	Coral_Recruits ~ Year	0.72	< 0.05
Artificial reef	Macroalgae ~ Year	0.99	< 0.05
Control reef	Urchins_Adult ~ Year	0.88	< 0.05
Control reef	Urchins_Juvenile ~ Year	0.83	< 0.05
Control reef	Urchins_Total ~ Year	0.96	0.60
Control reef	Coral_Recruits ~ Year	0.96	0.61
Control reef	Macroalgae ~ Year	0.93	0.20

The residuals for most models were non-normally distributed, therefore a non-parametric approach was selected.

**Table 2:** Ten Friedman tests were used to assess changes in juvenile, adult, and total *D. antillarum* population density, juvenile coral recruit abundance, and macroalgae cover over a three-year period.

Reef type	Predictor	Response variable
Control reef	Year (2015, 2016, 2017)	Juvenile *D. antillarum* abundance m^−2^
Control reef	Year (2015, 2016, 2017)	Adult *D. antillarum* abundance m^−2^
Control reef	Year (2015, 2016, 2017)	Total *D. antillarum* abundance m^−2^
Control reef	Year (2015, 2016, 2017)	Juvenile coral recruit abundance m^−2^
Control reef	Year (2015, 2016, 2017)	Macroalgae cover (%)
Artificial reef	Year (2015, 2016, 2017)	Juvenile *D. antillarum* abundance m^−2^
Artificial reef	Year (2015, 2016, 2017)	Adult *D. antillarum* abundance m^−2^
Artificial reef	Year (2015, 2016, 2017)	Total *D. antillarum* abundance m^−2^
Artificial reef	Year (2015, 2016, 2017)	Juvenile coral recruit abundance m^−2^
Artificial reef	Year (2015, 2016, 2017)	Macroalgae cover (%)

Changes in the value of each variable were assessed independently, and separate analyses were conducted for the control and artificial reefs.

## Results

### *D. antillarum* test diameters

**Figure 3:**
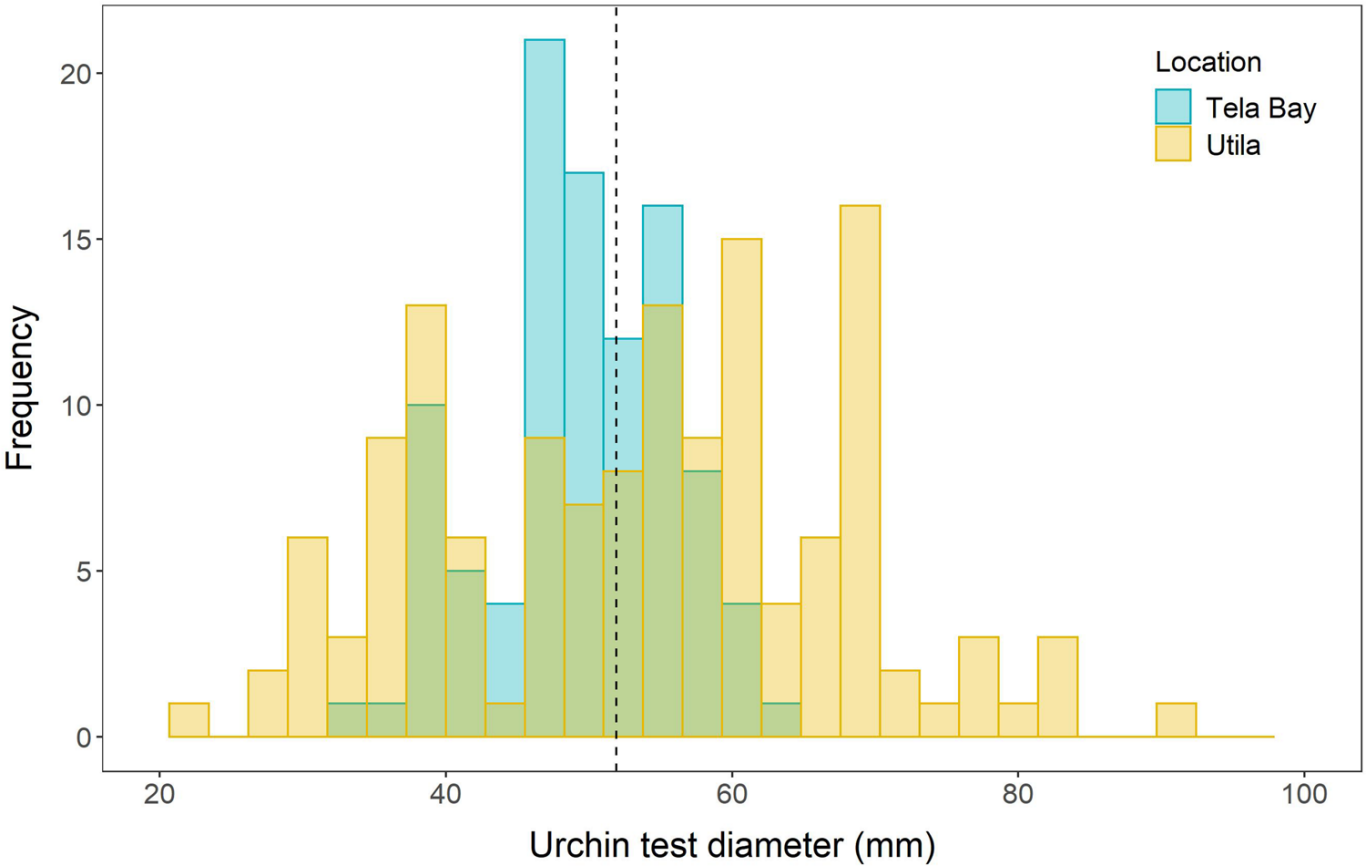
Distribution of adult *D. antillarum* test diameters on the reefs of Utila (yellow; *n* = 139) and Tela (blue; *n* = 100). Dotted vertical line is the mean of all individuals from both locations.

In total, 239 individual adult *D. antillarum* were collected from the reefs around Utila (*n* = 139) and Tela Bay (*n* = 100), and their test diameters were measured. At Utila, the mean test diameter was 53.52 ± 1.20 mm (mean ± 1SE) and the range was 21 to 90 mm. In Tela Bay, the mean was 49.69 ± 0.65 mm and the range was 32 to 64 mm (Fig. 3). The overall mean for both locations combined was 51.92 ± 0.76 mm. From this value it was inferred that the 5–15 cm spatial scale was the most ecologically relevant to *D. antillarum* based on need for the crevice to protect the test whilst providing additional space required for the articulated spines.

### In situ habitat preferences of *D. antillarum*

**Figure 4:**
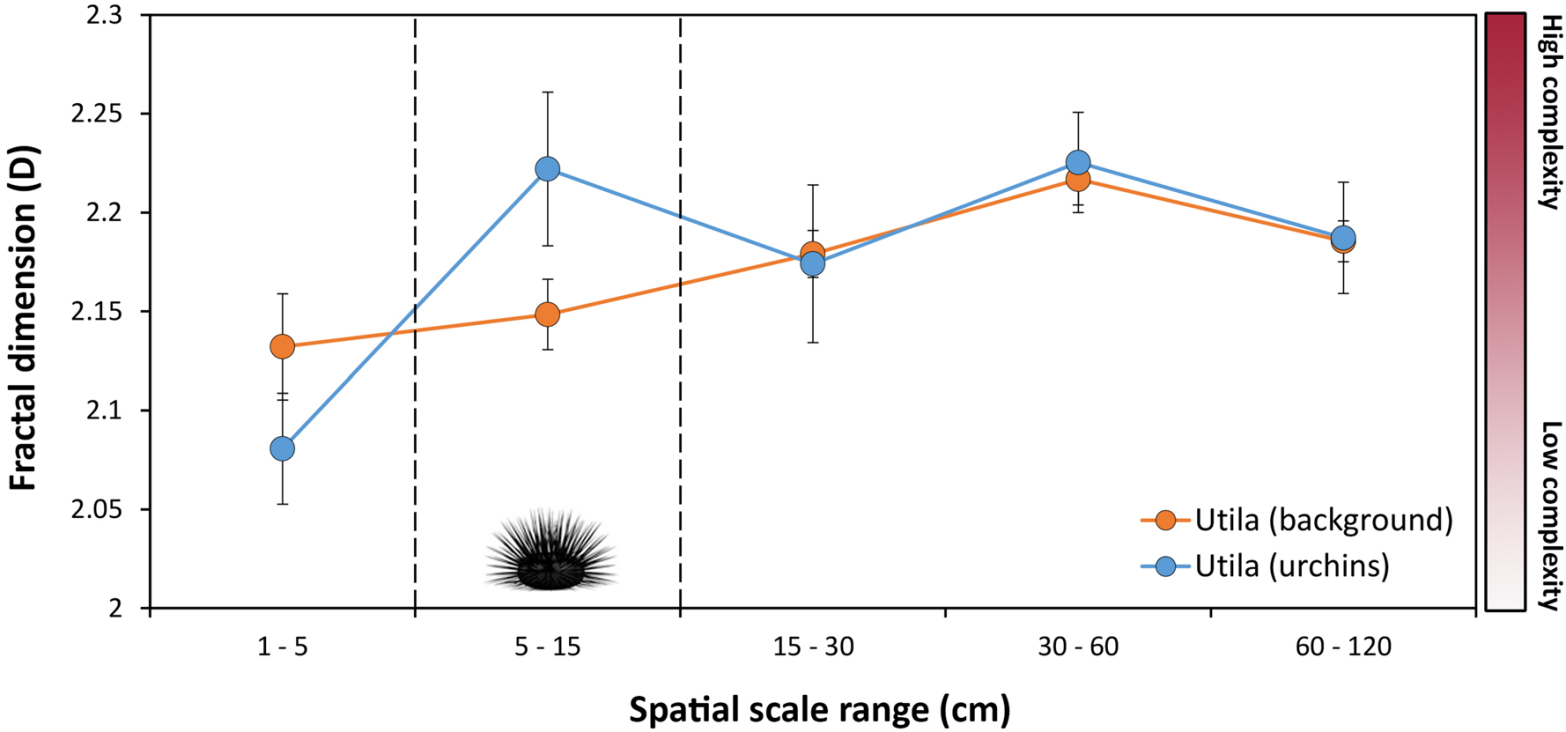
Complexity signatures of reef areas devoid of *D. antillarum* (orange, n = 35), and areas inhabited by *D. antillarum* (blue, n = 5). Data shown in the main panel are mean ± 1SE fractal dimension (D), a measure of structural complexity within defined spatial resolutions, shown at: 1–5 cm, 5–15 cm, 15–30 cm, 30–60 cm and 60–120 cm, with 5–15 cm representing the size range of ecological significance to *D. antillarum* as predation refugia. D ranges from 2 to 3 and higher values are associated with greater structural complexity. Error bars represent ± 1SE from the mean.

The habitat complexity (D) of areas inhabited by *D. antillarum* was compared to the mean complexity of uninhabited quadrats across five spatial scales; the only significant difference occurred at 5–15 cm, the range identified as most ecologically relevant to *D. antillarum* (Fig. 4). Within this size category, Utila’s reefs showed an average fractal dimension (D_5–15 cm_) of 2.14 ± 0.01 in uninhabited areas, while *D. antillarum* were found in areas with an average of 2.22 ± 0.04 (*W* = 38, *p* = 0.049). At spatial scales < 5 cm and > 15 cm, there were no differences in D between urchin-inhabited areas and urchin-uninhabited areas (15-30 cm: *W* = 98, *p* = 0.61; 30-60 cm: *W* = 80, *p* = 0.86; 60–120 cm: *W* = 94, *p* = 0.73).

### Impacts of structural complexity on predator avoidance behaviour

A two-way ANCOVA was used to assess the impact of structural complexity on the magnitude of *D. antillarum* Predator Avoidance behaviour, and it showed that the interaction between material and complexity was non-significant (*F*_1, 45_ = 0.69, *p* = 0.41). The resultant two-way ANOVA found that both complexity (*F*_1, 45_ = 62.26, *p* = 4.98^−10^) and material (*F*_2, 45_ = 13.52, *p* = 2.52^−5^) have a significant effect on the magnitude of *D. antillarum* PAB. However, post hoc Tukey–Kramer analysis revealed that material did not have an effect within the complexity treatments (Table 3).

**Table 3:** Predator avoidance behaviour (PAB) values (mean ± 1SE) under five different experimental treatments of habitat complexity and material used to create structure.

Complexity	Material	PAB (%)	Tukey–Kramer designation
Flat	Glass	54.84 ± 7.64	a
Low	Natural	39.22 ± 6.46	b
Low	Artificial	43.76 ± 8.04	b
High	Natural	23.86 ± 6.48	c
High	Artificial	24.79 ± 5.48	c

Tukey–Kramer designations show results of post hoc analysis of the two-way ANOVA to show where differences between treatments occur.

**Figure 5:**
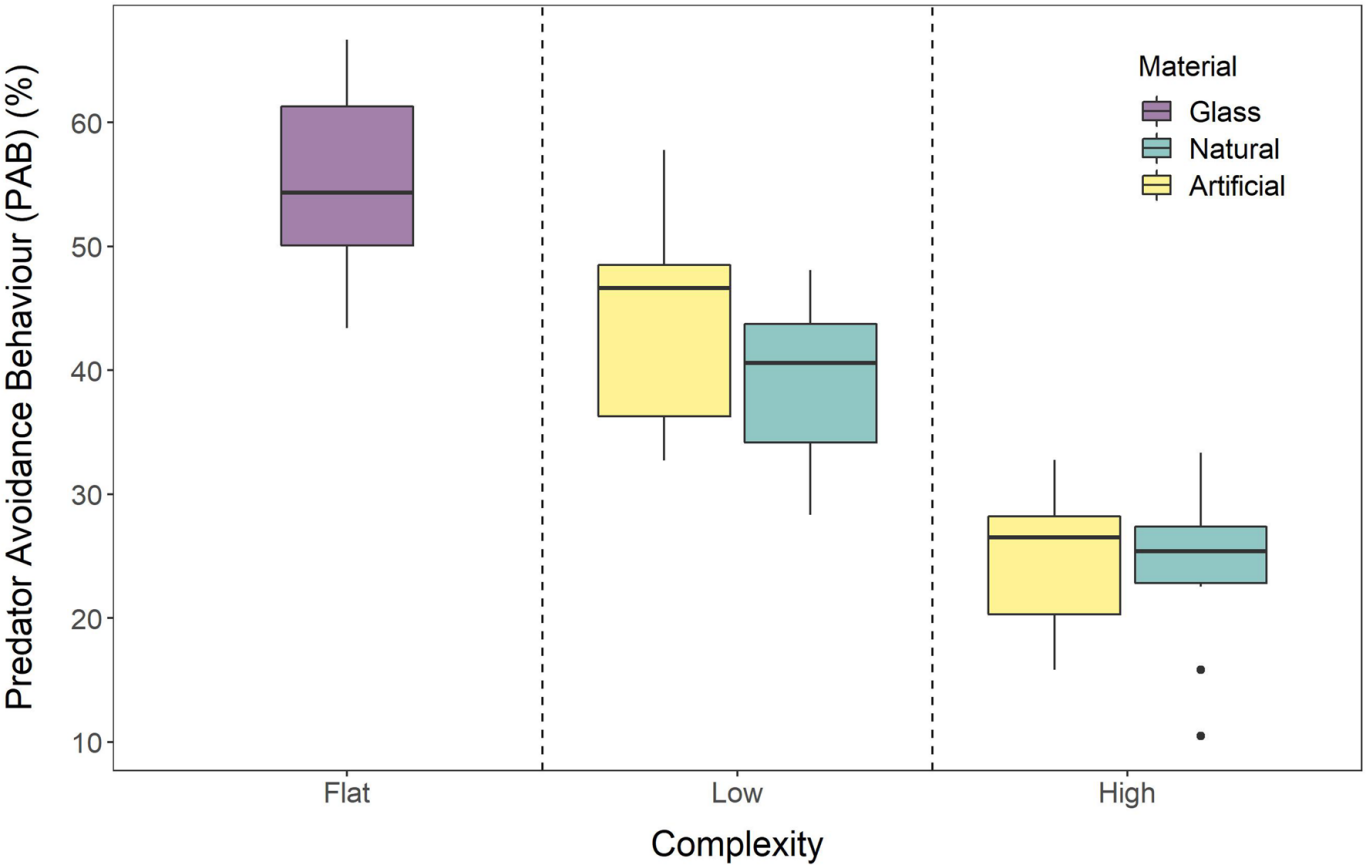
Impacts of habitat complexity on the predator avoidance behaviour (PAB) of *D. antillarum*. Flat treatments were carried out in trial tanks without any structure, low complexity treatments with natural material occurred in tanks enriched with fragments of reef rubble, and low complexity treatments with artificial materials were conducted in tanks with fragmented breezeblocks. High complexity treatments with natural material used reef rubble to simulate a reef crevice, and during high complexity treatments with artificial material individuals were provided with a whole breezeblock. The study was fully factorial and n = 10 for each treatment. The bold horizontal line on each boxplot represents the mean, the box itself shows the interquartile range and the whiskers delimit the full range of the data. Letters above the plots show the result of a post hoc Tukey–Kramer analysis and show where significant differences between treatments occur.

In the control treatment, where responses were tested in an unenriched tank with a glass bottom, mean PAB was 54.94 ± 7.64%. On average, PAB was reduced by 24% when the tank was enriched with a low complexity environment (natural material = 39.22 ± 6.46%; artificial material = 43.76 ± 8.04), and by 52% when it was enriched with a high complexity environment (natural material = 23.86 ± 6.48%; artificial material = 24.79 ± 5.48%) (Fig. 5).

### Impacts of artificial reefs on *D. antillarum* population density

At the start of this study, the degraded reefs of La Ensenada exhibited fractal dimension of the ecologically relevant 5–15 cm spatial range (D_5–15_) of 2.08 ± 0.01. This compared to 2.14 ± 0.01 for Utila’s reefs without urchins, and 2.22 ± 0.04 for Utila’s reefs with urchins. With the addition of our artificial reefs, this complexity was increased to 2.16 ± 0.00 (Fig. 2). Fractal dimension (D) used as a proxy for 3D structure gives a value between 2 (perfect 2-dimensional surface) to 3 (perfect 3-dimensional surface) therefore the 2.08 to 2.16 increase associated with the deployment of the artificial reefs represents a 100% increase in habitat complexity.

**Figure 6:**
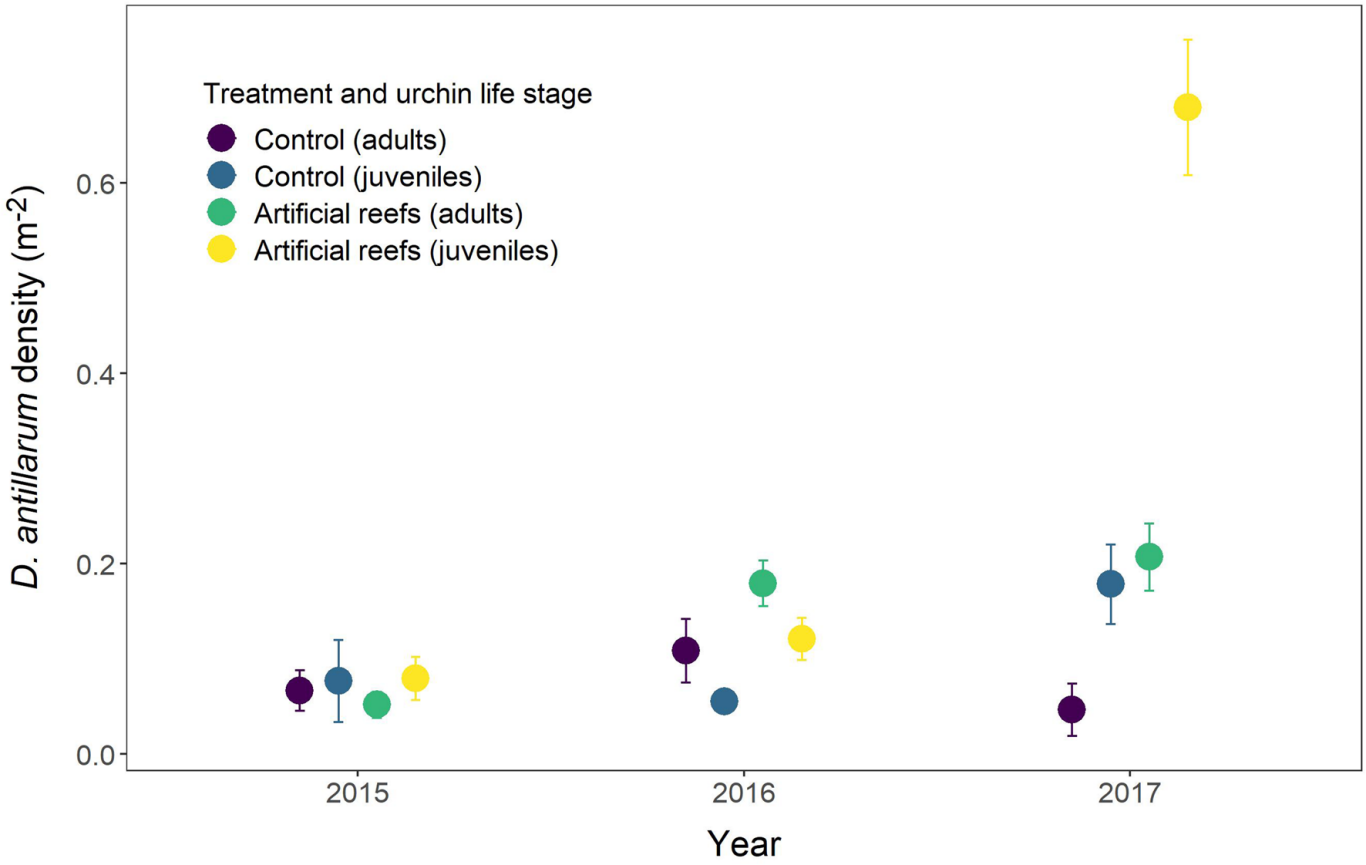
Temporal changes in the density of adult and juvenile *D. antillarum* on small artificial reef structures and nearby control reefs. Data were collected at the same time each year, but points are jittered to aid visualisation. Data shown are mean ± SE.

Prior to the deployment of ARs in 2015, control reefs in La Ensenada exhibited mean adult and juvenile *D. antillarum* densities of 0.07 ± 0.02 m^−2^ and 0.08 ± 0.04 m^−2^ respectively. There was no significant increase in adult *D. antillarum* population density over the study period (2016 = 0.11 ± 0.03 m^−2^; 2017 = 0.05 ± 0.03 m^−2^; *F*_2_ = 0.22, *p* = 0.64) (Fig. 6). However, whilst the juvenile *D. antillarum* population remained stable between 2015 and 2016, it had more than doubled by 2017 relative to the first time point (2016 = 0.06 ± 0.01 m^−2^; 2017 = 0.18 ± 0.04 m^−2^; *F*_2_ = 4.54, *p* = 0.05) (Table 4).

**Table 4:** Mean (SE) values of *D. antillarum* population densities (individuals m^−2^), macroalgal benthic cover (%) and juvenile coral recruit densities (individuals m^−2^) for control and artificial reefs in La Ensenada surveyed in 2015, 2016, and 2017.

	2015	2016	2017	*X*_2_	*p*
**Control reefs**					
Adult urchins (m^−2^)	0.07 (0.02)	0.11 (0.03)	0.05 (0.03)	0.22	0.64
Juvenile urchins (m^−2^)	0.08 (0.04)	0.06 (0.01)	0.18 (0.04)	4.54	0.05
Total urchins (m^−2^)	0.14 (0.06)	0.16 (0.04)	0.23 (0.05)	1.46	0.24
Macroalgae cover (%)	21.58 (0.50)	23.00 (2.53)	21.42 (2.36)	0.003	0.95
Coral recruits (m^−2^)	3.38 (0.28)	1.67 (0.22)	0.87 (0.15)	70.06	< 0.05
**Artificial reefs (ARs)**					
Adult urchins (m^−2^)	0.05 (0.01)	0.18 (0.02)	0.21 (0.04)	25.82	< 0.05
Juvenile urchins (m^−2^)	0.08 (0.02)	0.12 (0.02)	0.68 (0.07)	119.08	< 0.05
Total urchins (m^−2^)	0.13 (0.03)	0.30 (0.04)	0.89 (0.08)	85.01	< 0.05
Macroalgae cover (%)	56.07 (1.19)	52.92 (1.04)	38.13 (0.91)	74.19	< 0.05
Coral recruits (m^−2^)	4.32 (0.19)	3.47 (0.29)	4.01 (0.20)	4.38	< 0.05

Also shown are the results of Friedman tests comparing years.

Within a 5 m radius of the ARs, adult *D. antillarum* densities increased by 260% between 2015 (0.05 ± 0.01 m^−2^) and 2016 (0.18 ± 0.02 m^−2^), and by a further 17% to 0.21 ± 0.04 m^−2^ in 2017 (*X*_2_ = 21.20, *p* = 2.49 × 10^−5^) (Fig. 6). Changes in juvenile populations in the first year mirrored those of their adult counterparts (0.08 ± 0.02 to 0.12 ± 0.02 m^−2^), but a six-fold increase in density observed in the second year (to 0.679 ± 0.07 m^−2^) indicates a significant increase in the rate of recruitment (*X*_2_ = 116.15, *p* < 2.2 × 10^−16^) (Table 4).

### Benthic community changes on artificial reefs

Prior to AR deployment, control reefs exhibited mean macroalgae cover of 21.58 ± 0.50% and juvenile coral recruit densities of 3.38 ± 0.69 m^−2^ (Table 4). They showed no subsequent significant change in macroalgae cover over the course of this study, but the abundance of juvenile coral recruits decreased by 50% in the first year, and a further 47% in the second year (*X*_2_ = 12.00, *p* = 0.002).

By contrast, within a 5 m radius of the ARs, macroalgae cover decreased from 56.07 ± 1.19 to 52.92 ± 1.04% in the first year, and again to 38.13 ± 0.91% in the second year (*X*^2^ = 122.88, *p* < 2.2 × 10^−16^) representing a 17.94% reduction in actual percentage cover (Table 4). There was no significant change in the abundance of coral recruits throughout this time period, despite the significant decline on nearby control reefs.

## Discussion

Numerous correlative studies have suggested that *D. antillarum* populations are failing to recover because processes of reef flattening^8,39^ have reduced complexity creating a deficit of suitable habitat that leaves individuals vulnerable to predation^28,70^. The photogrammetric and lab-based data presented here provide experimental evidence to support this assertion. Fractal dimension analysis of 3D models^55^ demonstrates that *D. antillarum* preferentially inhabit areas of higher complexity (D) at ecologically relevant spatial scales. This indicates the existence of either an active preference for complexity, supported by studies showing that individuals compete for shelter even in the absence of a predation threat^71^, and/or a passive fitness benefit that allows recruits to mature beyond their predator escape size and therefore survive into adulthood^72,73^.

The importance of predation as a driver of *D. antillarum* population dynamics has been demonstrated experimentally in Puerto Rico, where the removal of the predatory wrasse species *Thalassoma bifasciatum* and *Halichoeres bivittatus* allowed significant population expansion^74^. This finding, coupled with the reduction in PAB associated with increasing structural complexity observed in the lab-based behaviour study, provides further evidence that the likely mechanism driving the high habitat complexity preference is the protection that complex habitats afford against predation. PAB is an important survival reflex, but it has a high energetic requirement and is therefore likely to be reduced in any scenario where the survival benefits do not outweigh the costs^48,59,60^, i.e. in a complex habitat where the defensive role of PAB is, at least partially, fulfilled by the individual’s environment.

As population densities increase, *D. antillarum* aggregate and form protective spine canopies^57^ which makes them less reliant on shelter for survival^75^; reliance on PAB is presumably also reduced. The importance of the interaction between habitat structure and predation as a driver of *D. antillarum* population dynamics is therefore likely to be greater in contemporary low-density populations than it was in historically high-density populations. Population restoration through provision of artificial shelter may therefore stimulate a positive feedback loop that ultimately reduces reliance on habitat structure and PAB. Artificial structures may protect individuals against predators, whilst also reducing reliance on PAB thus allowing more resources to be allocated to reproduction. These effects may operate synergistically to increase population density and allow the formation of a spine canopy, which would replace shelter and PAB to become the major mechanism of predatory defence. The low-habitat-complexity barrier to *D. antillarum* population recovery^28^ may be overcome.

It is also suggested that *D. antillarum* populations are failing to recover because they are asynchronous spawners^26,41,42^, which means they are unable to overcome the Allee effect that was established after their mass-mortality. This barrier is likely exacerbated by the loss of habitat that accompanied the functional extinction of *D. antillarum*^38^, as it increased nearest-neighbour distances and reduced the probability of fertilisation^40^. The significant PAB reduction observed in the high complexity treatment relative to the low complexity and flat treatments in the lab-based study may represent a large energy saving which allows individuals to allocate resources to reproduction rather than predatory defence^59^. Artificial reefs may therefore stimulate *D. antillarum* population recovery not only by reducing vulnerability to predation, but also by decreasing nearest-neighbour distances and increasing resource allocation to reproduction, both of which will help overcome the Allee effect that contributes to their continued suppression.

Data presented from the artificial reef (AR) study show that augmentation of habitat structure can stimulate increases in *D. antillarum* population density, and differences in the pattern of recovery between adults and juveniles corroborate the findings of earlier studies^26,40–42^ by indicating that adult densities must increase before juvenile recruitment can occur. The negative relationship between *D. antillarum* population density and macroalgae^15,21,22^ and the positive relationship between population density and juvenile coral recruits^76–78^ is well documented. It is therefore perhaps unsurprising, that our ecological surveys suggested that the colonisation of ARs by *D. antillarum* over the 24-months of this study stimulated both a significant decrease in macroalgae cover compared to adjacent control reefs, and maintained stable juvenile coral recruit densities against a backdrop of declining densities on control reefs. A longer time series would be needed to determine whether these initial benefits translate into longer-term increases in mature scleractinian coral cover.

The successes of *D. antillarum* restoration initiatives have been variable, but several studies in the Florida Keys and Jamaica have found that the ecological benefits can be profound^50,77,79^. A study that attempted to restock populations to mean densities of 4 individuals m^−2^ found that populations ‘relaxed’ to 1 individual m^−2^, and the high mortality was attributed to predation on reintroduced juveniles due to a lack of 3D structure on the reef^19^. This highlights that for reintroduction to be successful, the factor(s) preventing recovery must first be removed. The findings we present here suggest that the augmentation of structural complexity that accompanies the deployment of ARs may mitigate a major barrier to population recovery and increase the success of *D. antillarum* reintroduction initiatives, leading to improvements in reef health.

The three lines of experimental evidence presented here add weight to the previous assertion that habitat structure plays a key role in regulating *D. antillarum* population dynamics by creating predation refugia, and decreasing nearest-neighbour distances to increase the probability of fertilisation success^40,42^. Provision of artificial habitat complexity targeted towards the species-specific requirements of *D. antillarum* may stimulate population recovery on contemporary Caribbean coral reefs and reinstate the lost ecological functions they historically fulfilled to stimulate benthic recovery on degraded reefs.
